# 

**Published:** 1885-05

**Authors:** 


10 cts. a copy. MAY, 1885. $1.00 per Year.

### HALL'S JOURNAL OF HEALTH

ESTABLISHED 1854

OFFICE, 75 & 77 BARCLAY STREET, NEW YORK.

Entered as Second-class Matter at the Post-Office, New York.

